# Uric Acid Has Different Effects on Spontaneous Brain Activities of Males and Females: A Cross-Sectional Resting-State Functional MR Imaging Study

**DOI:** 10.3389/fnins.2019.00763

**Published:** 2019-07-26

**Authors:** Li Lin, Li Juan Zheng, U. Joseph Schoepf, Akos Varga-Szemes, Rock H. Savage, Yun Fei Wang, Han Zhang, Xin Yuan Zhang, Guang Ming Lu, Long Jiang Zhang

**Affiliations:** ^1^Department of Medical Imaging, Jinling Hospital, Medical School of Nanjing University, Nanjing, China; ^2^Division of Cardiovascular Imaging, Department of Radiology and Radiological Science, Medical University of South Carolina, Charleston, SC, United States

**Keywords:** uric acid, cognitive function, resting-stage functional magnetic resonance imaging, blood oxygen level dependent, amplitude of low-frequency fluctuation

## Abstract

**Objective:**

To explore the relationship among serum uric acid (SUA) levels in different states of disease, human cognition, and spontaneous brain activities by resting-state functional MRI (rs-fMRI).

**Methods:**

We prospectively recruited 100 subjects (age 58 ± 11 years, 55 females) who underwent fasting blood sampling, cognitive tests and rs-fMRI scans. The subjects were divided into two groups by sex and each sex group was further stratified into three subgroups according to SUA level in different states of disease. The amplitude of low-frequency fluctuation (ALFF) method was applied to assess spontaneous brain activity among groups. Pearson’s correlation analysis was used to investigate the relationships between the mean ALFF values (mALFF) and cognitive tests.

**Results:**

A total of 97 patients completed the study protocol successfully. Significant differences in age, education level, number connection test (NCT), and word fluency were observed among the three subgroups in males (all *P* < 0.05). Results of group-by-sex interaction were distributed in bilateral pallidum and putamen [voxel *P*-value < 0.001, cluster *P*-value < 0.05, Gaussian random field (GRF)-corrected]. The tendency of the SUA effect on mALFF was different in males and females, particularly in corresponding High SUA subgroups (that is pre-hyperuricemia, both *P* < 0.001). Among the male subjects, mALFF values of the bilateral pallidum and putamen negatively correlated with attention/executive function.

**Conclusion:**

Our results suggest that elevated SUA levels have different effects on spontaneous brain activities and cognitive function in males and females. Males with pre-hyperuricemia and hyperuricemia are more susceptible to changes in spontaneous brain activities and lower neuropsychological assessment scores, particularly in word fluency tests and NCT, compared to females.

## Introduction

Gout is a chronic and progressive disease caused by the deposition of monosodium urate crystals. The incidence of gout is growing from 0.30 per 1000 person-years to 2.68 per 1000 person-years from the 1970s to the 2000s (Dalbeth and Stamp, 2014). Comorbidities are common in patients with gout according to the 2007–2008 National Health and Nutrition Examination Survey data, such as hypertension and chronic kidney disease (Zhu et al., 2012; Dalbeth and Merriman, 2016). Gout is also associated with an increased risk of death, primarily due to cardiovascular disease (Choi and Curhan, 2007; Dalbeth and Merriman, 2016). Hyperuricemia, considered as stage A of gout in a new clinical staging system proposed in 2014, is the primary risk factor for gout (Dalbeth and Stamp, 2014). However, as serum uric acid (SUA) is an end product of purine metabolism and a natural antioxidant, it may also have neuroprotective effects (Khan et al., 2016). Consequently, more and more researchers have been committed to exploring the effect of SUA on cognitive function.

Previous studies found that patients with mild cognitive impairment and Alzheimer’s disease had lower SUA levels than healthy controls (Rinaldi et al., 2003; Kim et al., 2006). The Rotterdam study also found that individuals with higher SUA levels may have a lower risk of dementia and better cognitive function later in life (Euser et al., 2009). Recently, researchers studied the curative effect of SUA in acute stroke to confirm its neuroprotective effects. It has been suggested that SUA might reduce infarct growth and improve outcomes in patients with hyperglycemia during acute stroke (Amaro et al., 2015). However, other studies did not identify such neuroprotective effects related to SUA and even reported controversial results. One study found that there was no difference in outcome after stroke or safety concerns between the use of SUA in thrombolytic therapy or a placebo (Chamorro et al., 2014). Furthermore, another preclinical study found that SUA may induce hippocampal inflammation and may cause cognitive dysfunction in rodents and humans (Shao et al., 2016). In addition, elderly people with high SUA levels may have an increased risk of vascular or mixed dementia (Latourte et al., 2018), and mildly elevated SUA levels might increase the burden of cerebral ischemic pathology, particularly in older adults (Schretlen et al., 2007). In short, the neuroprotective effects of SUA are controversial and still uncertain.

Resting-state functional MRI (rs-fMRI), a sensitive and effective modality used to explore subtle brain function alterations, has been widely used in neuroimaging studies. The amplitude of low-frequency fluctuation (ALFF) is a reliable measure to detect the spontaneous neuronal activity in blood-oxygenation-level-dependent (BOLD)-related signals (Zhao et al., 2018). ALFF has been used in many neurodegenerative and neurometabolic disease studies, such as evaluating learning ability (Qian et al., 2016), consciousness level in acquired brain injury (Sharp et al., 2014; Zou et al., 2017), cognitive dysfunction in Alzheimer’s disease (Han et al., 2011; Wen et al., 2013), and schizophrenia (Meda et al., 2015; Hare et al., 2017). However, studies focusing on the effect of SUA levels on rs-fMRI-based cognitive function are warranted. Since hyperuricemia shows sex differences (Kuo et al., 2015; Dalbeth and Merriman, 2016), we hypothesize that SUA levels have different effects on cognitive function in males and females. Thus, this study aims to explore the effect of SUA levels and sex on spontaneous brain activity, as evaluated by ALFF, a rs-fMRI analytical algorithm.

## Materials and Methods

### Subjects

The study protocol was approved by the local Ethics Committee and all subjects provided written informed consent prior to the MRI and neuropsychological tests. This prospective study recruited 100 subjects from the local community. The inclusion criteria were as follows: right-handedness and aged 20–80 years without any history of psychiatric or neurological diseases. Participants with contraindications to MRI, history of gout, major adverse cardiovascular events, cerebrovascular disease, and severe chronic diseases with poor therapeutic control were excluded. Participants who had head motions with rotation of more than 1.0° or translation of more than 1.0 mm were excluded as well.

### Serum Assessment

Each subject underwent serum biochemical tests, including SUA, fasting plasma glucose (FPG), cholesterol (CHO), triglyceride (TRI), high-density lipoprotein cholesterol (HDL-C), low-density lipoprotein cholesterol (LDL-C), serum urea and serum creatinine. We estimated glomerular filtration rate (eGFR) using the Modification of Diet in Renal Disease (MDRD) equation to assess renal function (Poggio et al., 2005). Since SUA levels are consistently lower in females than in males (Rho et al., 2011; Latourte et al., 2018), subjects were grouped by sex. Then, each sex group was further stratified into three subgroups based on SUA levels: hyperuricemia level, high level (that is pre-hyperuricemia) and healthy level. Subgroups in males were: hyperuricemia level (Dis; SUA > 7.0 mg/dl); high level (High; SUA: 5.83–7.0 mg/dl); and healthy level (HC; SUA: 2.5–5.82 mg/dl), while subgroups in females were: Dis (SUA > 6.0 mg/dl); High (SUA: 5.0–6.0 mg/dl); and HC (1.66–5.0 mg/dl) (Chizyński, 2005).

### Neuropsychological Assessment

Neuropsychological assessment was conducted by a well-trained researcher (XX) with 5-year experience. Mini-Mental State Examination (MMSE) and Montreal Cognitive Assessment (MoCA) tests were used to assess general cognition status (Galea and Woodward, 2005; Nasreddine et al., 2005). Learning/memory and attention/executive function were assessed by vocabulary learning and word fluency tests, number connection test (NCT), and digit symbol test (DST). In addition, line tracing test (LTT), serial dotting test (SDT), and Stroop test have been applied to assess reaction capability (Su et al., 2016; Zheng et al., 2017).

### MR Imaging Data Acquisition

A 3-T MRI scanner (TIM Trio, Siemens Healthineers, Erlangen, Germany) equipped with the standard 12-channel head coil was used to scan all subjects. Participants were required to close their eyes and keep their head still during the scan (Su et al., 2016; Zheng et al., 2017). T2 fluid-attenuated inversion-recovery acquisition was used for clinically silent lesions screening with the following pulse sequence parameters: number of axial slices 25, slice thickness 4 mm, slice gap 1.2 mm, image matrix 232 × 256, field of view [FOV] 220 × 220 mm^2^, repetition time [TR]/echo time [TE] 9000 ms/93 ms, flip angle 130°, and inversion time 2500 ms. rs-fMRI data were acquired using a single-shot, gradient-recalled echo planar imaging sequence aligned along the anterior–posterior commissure to cover the whole brain for a period of 500 s, with the following parameters: number of volumes 250, image matrix 64 × 64, FOV 240 × 240 mm^2^, voxel size 3.75 × 3.75 × 4 mm^3^, number of axial slices 30, TR/TE 2000 ms/40 ms, and flip angle 90°. Lastly, anatomical images were obtained in the sagittal orientation using a high-resolution, T1-weighted, 3D, magnetization-prepared rapid gradient-echo sequence (T1 W-3D-MPRAGE) with the following parameters: TR/TE 2300 ms/2.98 ms, flip angle 9°, number of slices 191, FOV 256 × 256 mm^2^, acquisition matrix 256 × 256, and slice thickness 1 mm. The overall scan time was 20 minutes for each subject, and we inquired them after scan. All the subjects reported that they were not falling asleep during scanning.

### Data Preprocessing

The preprocessing was performed using Data Processing Assistant for Resting-State fMRI (DPARSF^1^ ; Yan and Zang, 2010; Yan et al., 2016). Initially, the first 10 volumes were deleted for both adjustment to the signal equilibrium and subjects’ adaptation to the environment. The images were then processed for slice timing and realignment. Subjects with translation or rotation parameters of more than 1.0 mm or 1.0° were excluded. Then, T1 W-3D-MPRAGE images were segmented into gray matter, white matter, and cerebrospinal fluid then normalized to the Montreal Neurological Institute space by using DARTEL (voxel size of 3 × 3 × 3 mm^3^). The T1 W-3D-MPRAGE images were thus co-registered to the fMRI data and smoothed with an isotropic Gaussian kernel of 6 mm. Finally, the processed data were detrended and nuisance covariates were regressed as well. Additionally, global signal regression was reported to reduce the relationships between motion and inter-individual differences in rs-fMRI metrics (Saad et al., 2012; Yan et al., 2012), we thus calculated the ALFF without global signal regression.

### ALFF Analysis

Amplitude of low-frequency fluctuation measures the amplitude of time series fluctuations at each voxel (Zhang et al., 2007), based on the DPARSF software. First, the time series for each voxel was filtered (band-pass, 0.01–0.1 Hz) and subsequently converted to the frequency domain by fast Fourier transform (FFT). The square root was calculated at each frequency of the power spectrum. The averaged square root was taken as the ALFF, and the mean ALFF was finally computed by the normalization of ALFF by the mean within-brain ALFF value for each subject.

### Statistical Analysis

The demographic and neuropsychiatric data analysis was performed with IBM SPSS Statistics 21.0. The Kolmogorov–Smirnov test was used to assess the normality of quantitative data. Normally distributed data were expressed as mean ± standard deviation. Then, the differences between the three subgroups, within the larger groups of males and females, were assessed by ANOVA test and the homogeneity of variance in these data was examined by the Levene test. The least significant difference (LSD) test was employed when the variance was homogeneous, while the Tamhane’s T2 was applied for the data with heterogeneous variance. Independent sample non-parametric test was used to analyze the data with non-normal distribution, which were reported as median and inter-quartile range [M(QU-QL)]. If the variance analysis revealed significant differences, *post hoc* analysis was conducted for inter-group comparisons. The differences between subgroups within each group were assessed by independent sample *t* test. *P* < 0.05 was regarded as a significant difference.

The rs-fMRI data were analyzed by Statistical Parametric Mapping 12 (SPM12)^2^. Primary analysis was assessed with a full factorial design (group-by-sex interactions, main effect of group, main effect of sex), with the covariance of age, education years, body mass index (BMI) and eGFR. The results were then corrected by Gaussian random field correction (GRF) (significance level was set at voxel *P* value < 0.001 and cluster *P* value < 0.05). *Post hoc* analysis was further performed with IBM SPSS Statistics 21.0 by extracting the values of brain regions with statistical differences using independent sample *t* test (between female and male with the corresponding group) and analysis of variance (among three subgroups respectively, in males and females). Significance level was set at *P* < 0.05. All the mALFF of the brain regions with significant differences were extracted for correlation analysis against the scores of the cognitive performance. Correlation analyses with IBM SPSS Statistics 21.0 were performed by using Pearson (normal distribution data) or Spearman correlation analysis (non-normal distribution data) with significant difference at *P* values < 0.05.

We calculated the power for two factor interactions (sex and uric acid levels) by using the G Power Software (version 3.1), which was described in detail in the Supplementary Material. In order to further study the effect of SUA concentration on the brain, we used SUA concentration as a continuous variable to study the interaction between sex and SUA concentration. Two sample *t* test design was used (SUA concentration-by-sex interactions, regression between SUA concentration and males/females), with the covariance of age, education years. The results were then corrected by the GRF (significance level was set at voxel *P* value < 0.001 and cluster *P* value < 0.05).

## Results

### Demographic and Neuropsychological Assessment Data

The demographics and neuropsychological assessment data are shown in Table 1. Subjects with head motions with translation of more than 1.0 mm or rotation of more than 1.0° (*n* = 2) and clinically silent lesions (posterior fossa cyst, *n* = 1) were excluded. Ninety-seven subjects including 55 females (mean age of 58 ± 11 years) and 42 males (mean age of 58 ± 14 years) were included in data analysis.

**TABLE 1:** Demographic and neuropsychological assessment data of the participants.

**Variables**	**Female**				**Male**			
	**Dis (*n* = 8)**	**High (*n* = 16)**	**HC (*n* = 31)**	***P***	**Dis (*n* = 15)**	**High (*n* = 9)**	**HC (*n* = 18)**	***P***
**Demographic data**								
Age (years)	61.13 ± 7.22	57.88 ± 8.65	56.48 ± 12.21	0.549^a^	51.20 ± 15.67	57.89 ± 15.99	63.39 ± 7.58	0.035^*a^
Education (years)	10.50 ± 2.27	9.44 ± 2.80	10.42 ± 3.43	0.563^a^	13.00 ± 3.21	9.22 ± 4.09	11.06 ± 2.98	0.031^*a^
BMI (kg/m^2^)	25.02 ± 2.32	24.68 ± 2.26	23.60 ± 2.57	0.203^a^	24.21 ± 2.50	25.92 ± 4.05	23.94 ± 2.88	0.270^a^
**Blood biochemical indices**								
FPG (mmol/L)	5.30 ± 0.75	5.05 ± 1.16	4.56 ± 0.93	0.096^a^	4.63 ± 0.96	4.34 ± 0.84	5.34 ± 1.38	0.070^a^
CHO (mmol/L)	5.54 ± 0.71	5.28 ± 1.05	4.81 ± 0.77	0.053^a^	4.60 ± 0.92	4.89 ± 0.61	4.54 ± 0.89	0.596^a^
TRI (mmol/L)	1.59 ± 1.11	1.70 ± 0.80	1.44 ± 1.07	0.696^a^	1.58 ± 1.38	1.44 ± 0.95	1.18 ± 0.65	0.538^a^
HDL-C(mmol/L)	1.35 ± 0.26	1.47 ± 0.18	1.49 ± 0.32	0.424^b^	1.33 ± 0.35	1.13 ± 0.23	1.28 ± 0.29	0.273^a^
LDL-C(mmol/L)	3.63 ± 0.80	3.14 ± 0.81	2.69 ± 0.62	0.003^*⁣*a^	2.65 ± 0.67	3.06 ± 0.81	2.73 ± 0.73	0.393^a^
Scr (μmol/L)	66.61 ± 11.56	52.34 ± 7.67	51.60 ± 10.73	0.002^*⁣*a^	75.07 ± 9.44	72.02 ± 11.17	70.76 ± 10.08	0.473^a^
eGFR (ml/min/1.73 m^2^)	97.00 ± 17.00	131.38 ± 23.28	137.13 ± 23.03	0.001^*⁣*a^	109.67 ± 20.92	113.78 ± 25.97	113.17 ± 23.03	0.880
**Neuropsychological assessment**								
MMSE	26.88 ± 2.42	27.88 ± 1.89	27.87 ± 1.78	0.401^a^	28.67 ± 1.35	27.22 ± 1.72	27.78 ± 1.44	0.061^a^
MoCA	23.75 ± 3.77	24.94 ± 3.19	25.35 ± 2.69	0.408^a^	25.93 ± 2.96	26.11 ± 3.41	25.17 ± 2.46	0.642^a^
Vocabulary learning (*n*)	14.63 ± 3.96	16.50 ± 5.62	17.87 ± 5.74	0.309^a^	17.73 ± 6.43	13.78 ± 10.11	15.72 ± 4.57	0.373^b^
Delayed recall (*n*)	3.88 ± 3.00	4.69 ± 2.55	5.23 ± 2.57	0.413^a^	5.40 ± 3.20	4.44 ± 3.54	5.22 ± 2.00	0.712^a^
NCT(s)	84.50 ± 88.13	55.69 ± 23.39	64.83 ± 39.05	0.355^b^	42.80 ± 17.29	73.78 ± 27.11	73.44 ± 49.51	0.042^*a^
DST(*n*)	32.50 ± 13.47	37.25 ± 13.10	43.29 ± 21.66	0.277^a^	46.60 ± 14.43	38.56 ± 23.17	36.11 ± 17.50	0.242^a^
LTT(s)	57.25 ± 16.28	45.75 ± 21.56	54.26 ± 40.87	0.645^a^	43.20 ± 24.42	48.00 ± 11.34	48.28 ± 21.88	0.767^a^
SDT(s)	55.13 ± 12.17	46.06 ± 14.15	51.06 ± 20.06	0.458^a^	38.47 ± 10.59	49.78 ± 17.25	51.33 ± 23.82	0.133^a^
Word fluency (*n*)	3.50 ± 2.51	4.44 ± 2.76	3.74 ± 2.41	0.596^a^	4.93 ± 1.98	2.78 ± 2.17	5.61 ± 2.99	0.029^*a^
StroopI(s)	18.88 ± 7.79	18.06 ± 7.16	19.45 ± 7.79	0.839^a^	23.20 ± 9.88	23.67 ± 8.80	19.61 ± 7.53	0.388^a^
StroopII(s)	24.63 ± 7.37	26.00 ± 6.46	24.42 ± 8.25	0.764^a^	29.87 ± 8.63	29.11 ± 9.44	27.56 ± 9.16	0.758^a^
StroopIII(s)	30.75 ± 11.02	32.25 ± 7.75	32.16 ± 9.46	0.920^a^	38.20 ± 8.93	38.00 ± 10.20	36.00 ± 14.41	0.847

Mean ± standard deviation; s, second; *n*, number; MMSE, Mini-Mental State Examination; MoCA, Montreal Cognitive Assessment; NCT, number connection test; DST, digit symbol test; LTT, line tracing test; SDT, serial dotting test; FPG, Fasting plasma glucose; CHO, cholesterol; TRI, triglyceride; HDL-C, High-density lipoprotein cholesterol; LDL-C, Low-density lipoprotein cholesterol; Scr, serum creatinine; eGFR, estimated glomerular filtration rate; ^*^P < 0.05, ^∗∗^*P* < 0.01; ^*a*^ LSD(L); ^*b*^Tamhane’s T2(M).

There were no significant differences in age, BMI, education level or neuropsychological assessment among the three subgroups of females (all *P* > 0.05). However, there were significant differences in age, education level, NCT, and word fluency among the three subgroups of males. Specifically, the differences of age and NCT were shown between Dis and HC subgroups (*P* = 0.037, *P* = 0.021, respectively), and education years were different between Dis and High subgroups (*P* = 0.021). The word fluency tests showed significant difference between Dis versus High subgroups and High versus HC subgroups (*P* = 0.048; *P* = 0.009, respectively).

For blood biochemical indices, there were no differences among subgroups of males and females (all *P* > 0.05), except for LDL-C, Scr and eGFR in females (*P* = 0.003).

### Results of ALFF Analysis

#### Group-By-Sex Interaction

**FIGURE 1:**
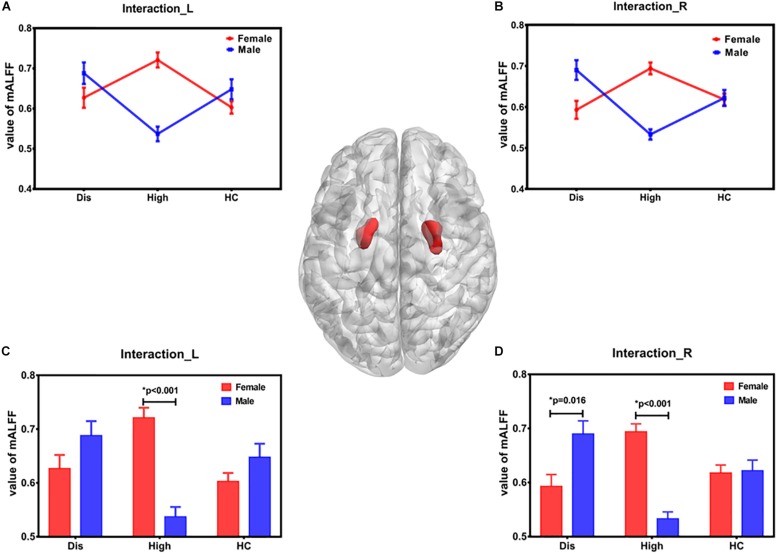
Brain regions with statistical differences in group-by-sex interaction. The brain regions influenced by group-by-sex interaction was bilateral pallidum and putamen, shown in the middle brain figure. Panels **(A,B)** show the tendency of SUA effect on ALFF in males and females. In females, the mean ALFF value of High subgroup was the highest, while the lowest in males. Panels **(C,D)** display the mALFF difference between females and males in each SUA level subgroup. *L* – left, *R* – right, *F* – female, *M* – male, *SUA* – serum uric acid, *mALFF* – mean value of ALFF, *Dis* – hyperuricemia level, *High* – high level, *HC* – healthy level.

The results of group-by-sex interaction and its *post hoc* analysis is shown in Figure 1 and Table 2. Our study found that the mALFF in bilateral pallidum and putamen was modulated (GRF correction, *P* value < 0.001 at voxel level and *P* value < 0.05 at cluster level). The tendency of SUA effect on mALFF was different in males and females (Figures 1A,B). Specifically, as for the sex difference with the corresponding SUA level, the mALFF of bilateral pallidum and putamen were different in the High group (both *P* < 0.001), while only the mALFF of the right pallidum and putamen were different in the Dis group (*P* = 0.005) (Figures 1C,D).

**TABLE 2:** Differences of mALFF among groups.

**Contrast**	**Region (AAL)**	**Cluster**	**MNI coordinates**			**Peak**
		**size**	**(mm)**			***f* value**
			***x***	***y***	***z***	
**Group-by-Sex Interaction**						
	Pallidum and Putamen (L)	35	–24	–6	6	12.898
	Pallidum and Putamen (R)	37	27	0	0	12.897

*All voxel *P* value < 0.001, cluster *P* value < 0.05, with GRF corrected; *L* – left, *R* – right.*

**FIGURE 2:**
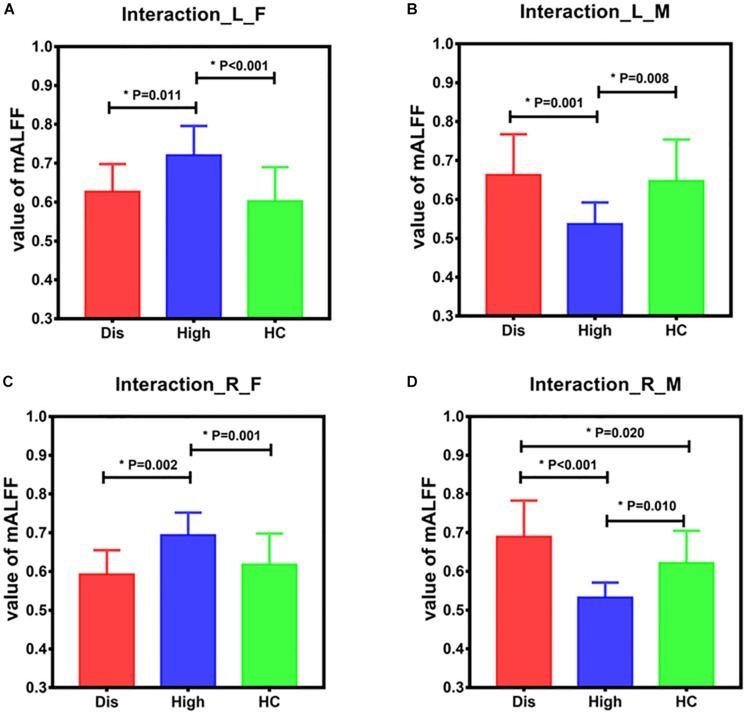
Differences of brain regions influenced by group-by-sex interaction among the three subgroups for male and female. Panels **(A,B)** show the mean ALFF value extracted from left pallidum and putamen among three subgroups for females and males. Panels **(C,D)** show the mean ALFF value extracted from right pallidum and putamen among three subgroups for females and males. In females, the differences are shown in bilateral pallidum and putamen between Dis and High subgroups and between High and HC subgroups. In males, there are differences in bilateral pallidum and putamen between Dis and High subgroups and between High vs. HC subgroups, and differences in right pallidum and putamen between Dis vs. HC subgroups. *L* – left, *R* – right, *F* – female, *M* – male, *mALFF* – mean vale of ALFF, *Dis* – hyperuricemia level, *High* – high level, *HC* – healthy level.

The differences among the three subgroups of males and females are shown in Figure 2. In females, the differences were shown in the bilateral pallidum and putamen between Dis vs. High subgroups (Dis < High, right: *P* = 0.001; left: *P* = 0.018) and between High vs. HC subgroups (High > HC, right: *P* = 0.001; left: *P* < 0.001) (Figures 2A,C). In males, the differences of the bilateral pallidum and putamen were between Dis vs. High subgroups (Dis > High, right: *P* < 0.001; left: *P* = 0.001) and High vs. HC subgroups (High < HC, right: *P* = 0.004; left: *P* = 0.006), and differences in the right brain regions between Dis vs. HC subgroups (Dis > HC, *P* = 0.014) (Figures 2B,D).

The correlation analysis between brain regions with statistical differences among groups and neuropsychological assessment is described in Table 3. For male subjects, the mALFF values of bilateral pallidum and putamen negatively correlated with the NCT scores (right, *P* = 0.027, *R* = –0.342; left, *P* = 0.039, *R* = –0.320), while the mean ALFF values of left pallidum and putamen positively correlated with the scores of word fluency (*P* = 0.004, *R* = 0.439). The mean ALFF values of bilateral pallidum and putamen were not correlated with scores of any neuropsychological tests across female subjects.

**TABLE 3:** Correlation Analysis between brain regions with statistical differences of interaction and neuropsychological assessment data.

**Variables**	**Pallidum and Putamen _L**				**Pallidum and Putamen _R**			
	**Female**		**Male**		**Female**		**Male**	
	***P* value**	***R* value**	***P* value**	***R* value**	***P* value**	***R* value**	***P* value**	***R* value**
MMSE	0.684	0.056	0.103	0.255	0.770	0.04	0.054	0.299
MoCA	0.330	–0.134	0.188	0.207	0.432	–0.108	0.973	–0.005
Vocabulary learning (*n*)	0.181	–0.183	0.567	0.091	0.201	–0.175	0.380	0.139
Delayed recall (*n*)	0.361	–0.126	0.775	–0.045	0.557	–0.081	0.626	0.077
NCT (s)	0.680	–0.057	0.039^*^	–0.320	0.455	–0.104	0.027^*^	–0.342
DST (*n*)	0.431	–0.108	0.254	0.180	0.690	–0.055	0.236	0.187
LTT (s)	0.505	–0.092	0.185	–0.208	0.321	–0.136	0.109	–0.251
SDT (s)	0.125	–0.209	0.103	–0.255	0.062	–0.253	0.052	–0.302
Word fluency (*n*)	0.565	0.079	0.004^*^	0.439	0.542	0.084	0.134	0.235
StroopI(s)	0.678	0.057	0.706	0.06	0.452	0.104	0.742	0.052
StroopII(s)	0.574	0.078	0.766	–0.047	0.738	0.046	0.799	0.040
StroopIII(s)	0.777	–0.039	0.101	–0.257	0.638	–0.065	0.507	–0.105

s = second; *n* = number; MMSE = Mini-Mental State Examination, MoCA = Montreal Cognitive Assessment, NCT = number connection test, DST = digit symbol test, LTT = line tracing test, SDT = serial dotting test; ^*^P < 0.05, ^∗∗^*P* < 0.01.

### Main Effect of Sex and Group

**FIGURE 3:**
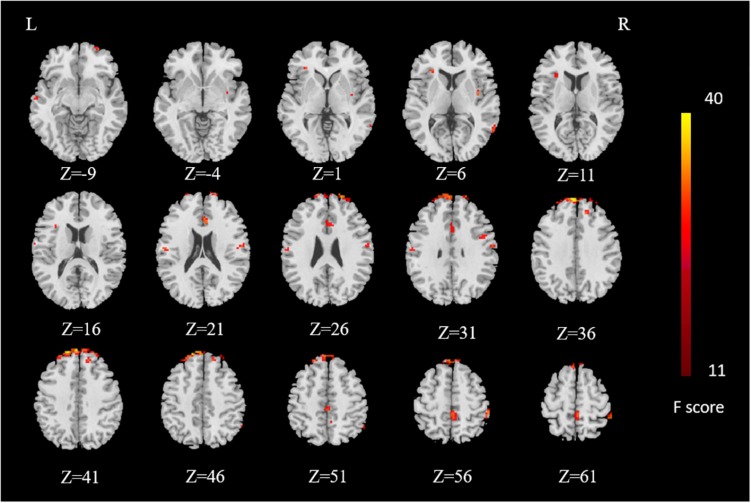
Results of main effect of sex. Brain regions influenced by main effect of sex are distributed in superior frontal gyrus, anterior cingulate, paracentral lobule in bilateral cerebrum, inferior frontal gyrus, middle temporal gyrus, precentral gyrus in right cerebrum, transverse temporal gyrus, and postcentral gyrus in left cerebrum. The *F* value color-coded scale is reported at the right side of the images. *L* – left, *R* – right.

Brain regions influenced by the main effect of sex were distributed in superior frontal gyrus, anterior cingulate cortex, paracentral lobule in bilateral cerebrum, inferior frontal gyrus, middle temporal gyrus, precentral gyrus in right cerebrum, transverse temporal gyrus, and postcentral gyrus in left cerebrum (voxel *P* value < 0.001, cluster *P* value < 0.05, GRF-corrected) (Figure 3). However, we did not find any brain regions influenced by the main effect of group after GRF correction.

### Result of Secondary Analysis

We used SUA as a continuous variable to study SUA concentration-by-sex interactions. There was no cluster survived after GRF correction. Furthermore, there was no cluster survived after GRF correction neither in males or females, when we did regression between SUA concentration and males/females, respectively.

## Discussion

This study analyzed the potential association between SUA in different states of disease and spontaneous brain activities evaluated by rs-fMRI. We found different patterns of spontaneous brain activities in males and females with different SUA levels in different states of disease. Our results suggest that elevated SUA levels are associated with lower neuropsychological assessment scores, particularly in word fluency tests and NCT. Furthermore, changes in spontaneous brain activities and cognition assessment scores appeared before hyperuricemia, and males were more susceptible than females. These findings shed a new light on cognitive function changes influenced by the different SUA levels.

Our study showed that the changes in spontaneous brain activities were mainly in the pallidum and putamen, which were correlated with word fluency and NCT scores. Pallidum and putamen are the structures that compose the basal ganglia. The putamen plays an important role in several basal ganglia–thalamocortical circuits (Alexander et al., 1986) involved in motor control and learning, as well as selecting and enabling cognitive, executive, and emotional programs (Ring and Serra-Mestres, 2002). Putamen has also been found to play a key role in the pathophysiology of patients with attention-deficit/hyperactivity disorder (Greven et al., 2015). Consistently, our study showed that the mALFF of the pallidum and putamen was positively correlated with word fluency and negatively correlated with NCT. These findings indicate that the results were closely related to attention/executive function. Moreover, since a lower score of NCT and a higher score of word fluency stand for better attention/executive function, we speculate that subjects with a higher mALFF might have better attention/executive function.

We also found that the High subgroup of males had the lowest mALFF and performed worst in word fluency tests and NCT. However, we did not observe this finding in females. This suggests a possible sex-related effect of SUA on attention/executive function. Our study thus implies that attention/executive function might be impaired in males with pre-hyperuricemia, in accordance with previous studies (Vannorsdall et al., 2008; Shao et al., 2016; Desideri et al., 2017). One previous study found that female hormones are associated with lower SUA levels through renal clearance (Hak and Choi, 2008), the possible reason might be a relative protective effect of estrogen (Mauvais-Jarvis, 2015; Dekkers et al., 2019).

Vannorsdall et al. (2008) found that high SUA levels were associated with greater white matter hyperintensities and poorer working memory, processing speed, fluency, and verbal memory. Another study suggested that an increased SUA level may result in cognitive dysfunction by causing hippocampal inflammation (Shao et al., 2016). Furthermore, an *in vitro* experimental model documented that high SUA levels could exert detrimental effects on brain structure and function by directly influencing the viability of neuronal cells and their ability to establish synaptic connections (Desideri et al., 2017). Importantly, our investigation, in agreement with the study by Baena et al. (2017), found a possible sex-related effect of SUA on executive function in a middle-aged population. However, Baena et al. (2017) found better cognitive performance in an executive function test in men but not in women, while our results showed the attention/executive function impaired in the High subgroup of males. Possible explanation for the difference is that the high SUA level of their subjects might be in compensatory state, and the results were just like what we found in Dis level. Furthermore, we did not find any brain regions with statistical difference by secondary analysis, which corresponds with the results of primary analysis as the trend showed in Figures 1A,B.

All the alterations indicated the sensitivity and vulnerability of the mALFF derived from the period before the hyperuricemia. However, the European League Against Rheumatism and American College of Rheumatology recommend reducing SUA level in the state of hyperuricemia (Richette et al., 2017). According to our current study findings, it is rational to pay more attention to the subjects with pre-hyperuricemia rather than one with hyperuricemia. However, more clinical studies are needed to validate our hypothesis.

Our study has some limitations. First, the sample size was small, thus it is necessary to further validate our findings in a larger cohort. Second, subjects in the different subgroups were not matched. Third, we only studied the difference of ALFF among SUA levels, and more brain function indices should be used to confirm our findings. Fourth, our study is a cross-sectional study, which could not show cognitive function changes during the process of disease progression. Fifth, subjects with their eyes closed in the scanner may not be awake all the time, although we collected rs-fMRI data at first, and told the subject not to fall asleep before scanning, in order to reduce the probability of the subject falling asleep. In future study, we will add some objective indicators to monitor the state of subjects during scanning, such as electroencephalogram. Last but not least, although we controlled for many cardiovascular disease risk factors, there are many other confounding factors as well. We could not exclude the possibility that SUA affected cognitive function by increasing the risk of cardiovascular disease. More work should be done to confirm our findings in the future.

In conclusion, our results suggest that elevated SUA levels were associated with lower neuropsychological assessment scores, particularly in word fluency tests and NCT. Furthermore, changes in spontaneous brain activities and cognition appeared before hyperuricemia, and males are more susceptible than females. This is very implicative for the clinical management for patients with pre-hyperuricemia and hyperuricemia although further clinical studies are urgently needed to demonstrate this finding.

## Ethics Statement

The study protocol was approved by the local Ethics Committee and all subjects provided written informed consent prior to the MRI and neuropsychological tests.

## Author Contributions

All authors listed have made a substantial, direct and intellectual contribution to the work, and approved it for publication.

## Conflict of Interest Statement

UJS is a consultant for and/or receives research support from Astellas, Bayer, Elucid BioImaging, General Electric, Guerbet, HeartFlow, and Siemens Healthineers. AV-S receives institutional research support from Siemens. The remaining authors declare that the research was conducted in the absence of any commercial or financial relationships that could be construed as a potential conflict of interest.

## Footnotes

1 http://rfmri.org/DPARSF

2 http://www.fil.ion.ucl.ac.uk/spm/
