# Supplementary material for: Uric Acid Has Different Effects on Spontaneous Brain Activities of Males and Females: A Cross-Sectional Resting-State Functional MR Imaging Study
Source: Front Neurosci. 2019 Jul 26;13:763. doi: 10.3389/fnins.2019.00763 (PMC6676594; doi:10.3389/fnins.2019.00763)
Supplement: Supplementary file 1 [file Table_1.DOCX]

**Supplementary Material**

We calculated the power for two factor interactions (gender and uric acid levels) by using the G Power Software (version 3.1). The method of power analysis we used was Fixed effects ANOVA - special, main effects and interactions. The type of power analysis was set to Post hoc. We first calculated the effect size f corresponding to our data. Then we used G * Power to calculate the power for α= 0.05 and a total sample size = 97. The parameter we set were as follow:

**F tests -** ANOVA: Fixed effects, special, main effects and interactions

**Analysis:** Post hoc: Compute achieved power

**Input:** Effect size f = 0.38

α err prob = 0.05

Total sample size = 97

Numerator df = 5

Number of groups = 6

**Output:** Noncentrality parameter λ = 14.0068000

Critical F = 2.3145472

Denominator df = 91

Power (1-β err prob) = 0.8124893

As results presented above, by using a sample size of 97, we can reach a power of 0.8124893 for α= 0.05.
